# Treatment modalities and patient reported outcomes in isolated and combined dorsal triquetrum chip fractures

**DOI:** 10.1007/s00068-025-02947-y

**Published:** 2025-08-22

**Authors:** Silvan Pasquinelli, Timo Tondelli, Sebastian Guenkel

**Affiliations:** 1 Department of Orthopaedics and Trauma Surgery, Bürgerspital Solothurn, Solothurn, 4500 Switzerland; 2 Department of Hand Surgery, University Hospital Bern - Inselspital, Freiburgstrasse 20, Bern, 3010 Switzerland; 3 Department of Orthopaedics and Trauma Surgery, University Hospital Basel, Basel, 4031 Switzerland; 4 Department of Orthopaedics and Trauma Surgery, Zürich City Hospital - Triemli, Zürich, 8063 Switzerland

**Keywords:** Wrist, Triquetrum, Fracture, Treatment

## Abstract

**Purpose:**

Triquetrum fractures are the second most common carpal fracture after scaphoid fractures with dorsal triquetrum chip fracture being the most common fracture type. There is little evidence about treatment and outcome of these fractures.

**Method:**

In this retrospective study of 39 patients, non-operative treatment with a wrist orthosis is compared to a cast. Furthermore, the duration of immobilisation is analysed. Incidence and outcomes of isolated dorsal triquetrum chip fracture and in combination with concomitant ipsilateral upper limb injuries were studied.

**Results:**

We found no difference in patient reported outcome measures, range of motion (ROM) and function for isolated dorsal triquetrum fractures in terms of type and time of immobilisation. Conservative treatment of these fractures resulted in good overall outcomes (PROMs, ROM), whereby ROM was slightly better for isolated injuries compared to combined injuries.

**Conclusion:**

In general, these fractures can be treated non-operatively with favourable patient-reported and clinical outcomes regardless of immobilisation modality and duration.

Level of evidence III (therapeutic).

## Introduction

The Os triquetrum is part of the proximal row of carpal bones. It is pyramidally shaped and almost completely covered with cartilage. Its palmar surface articulates with the Os pisiforme. Proximally it articulates with the TFCC, distally with the Os hamatum, and radially with the Os lunatum.

Triquetral fractures account for 19% of carpal fractures [1]. Thus, they are considered the second most common fractures of the carpal bones after scaphoid fractures [1–4].

Three types of triquetrum fractures can be distinguished: Dorsal triquetrum chip (dorsal cortical) fractures form the largest proportion [3]. The causing trauma mechanisms remains unclear [5]. Triquetral body fractures are usually associated with higher energy traumas and occur e.g. in perilunate dislocation injuries. Volar cortical fractures correspond to osseous avulsions of the palmar portion of the lunotriquetral ligament (LTIO).

Treatment of dorsal triquetrum chip fractures is usually conservative using cast immobilisation for 4–6 weeks. Höcker et al. found in their series with 65 followed patients very good results in 86% of patients after 4 years of follow-up [3]. However, the necessary duration of immobilisation remains unclear in present literature [2].

No clear therapeutic guidelines exist for corpus fractures. Nondisplaced fractures of the corpus can be treated by cast immobilisation for 4–6 weeks [6]. Osteosynthesis of corpus fractures is rarely necessary, but cases have been described [7]. For triquetrum fractures in perilunate dislocation injuries, the focus is on fixation of the LTIO using pins and not primarily fracture care itself.

**Fig. 1:**
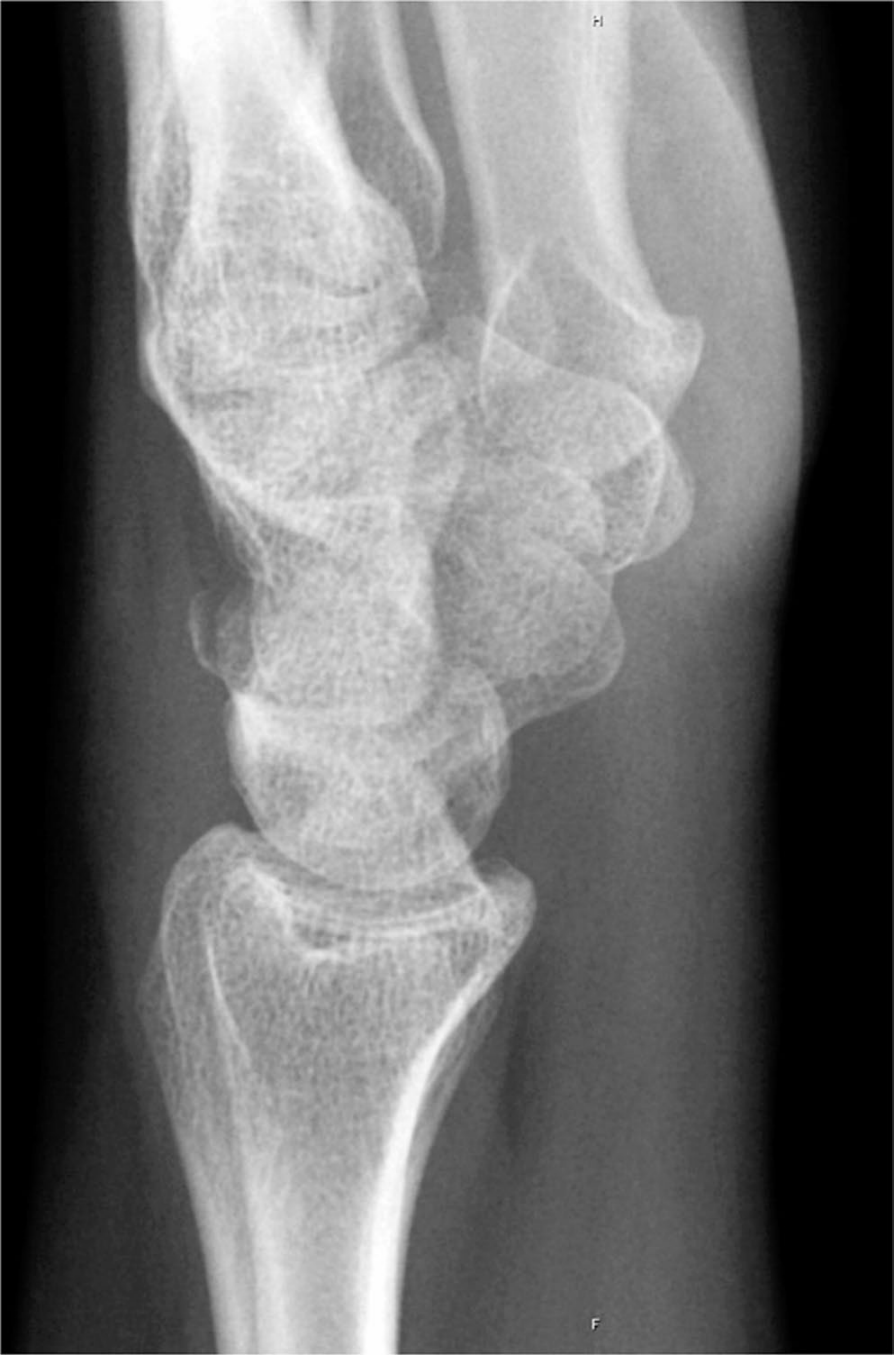
In a lateral wrist x-ray view, a small cortical fragment of the triquetrum is normally seen

**Fig. 2:**
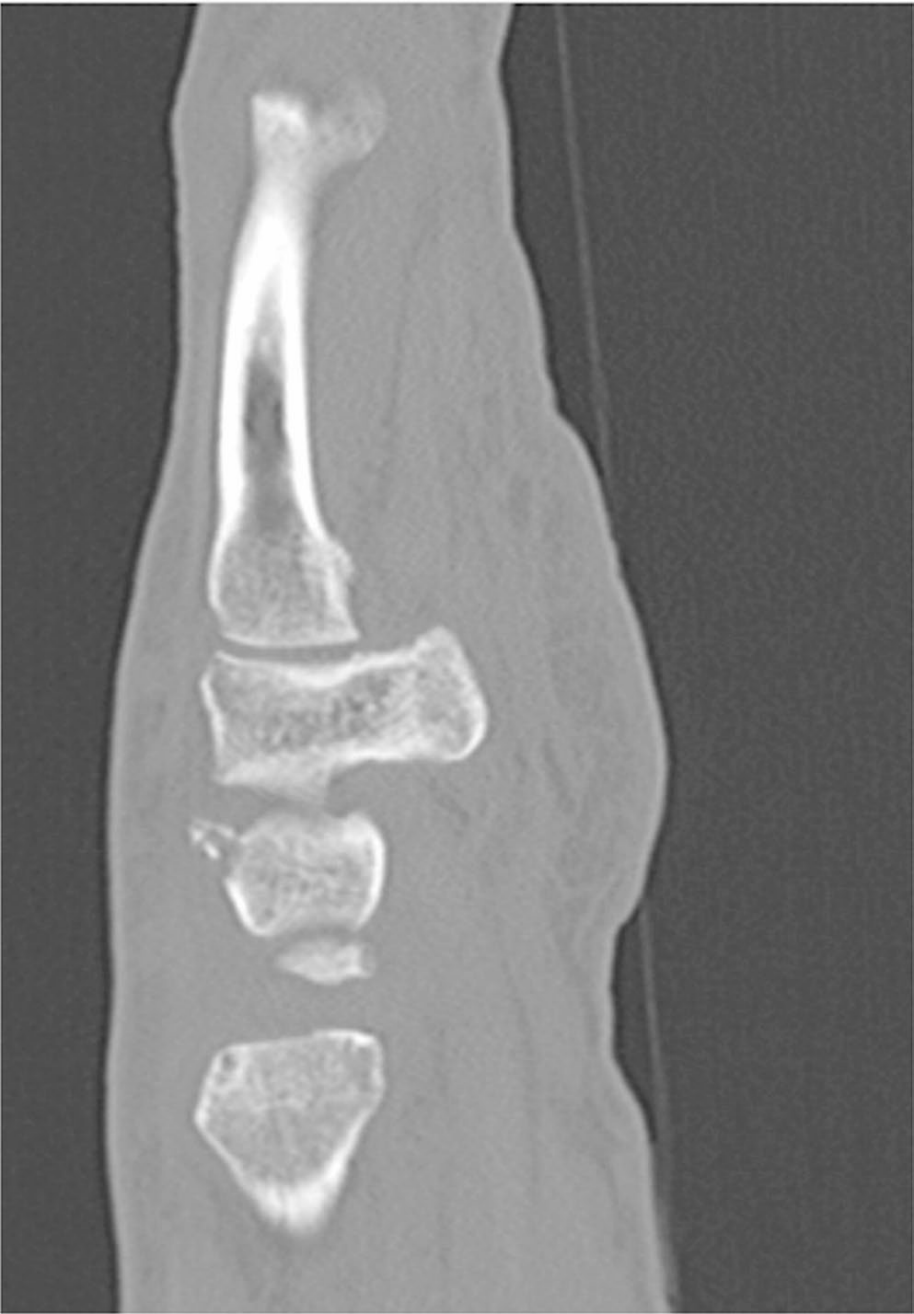
Typical CT scan findings in a sagittal reconstruction

**Fig. 3:**
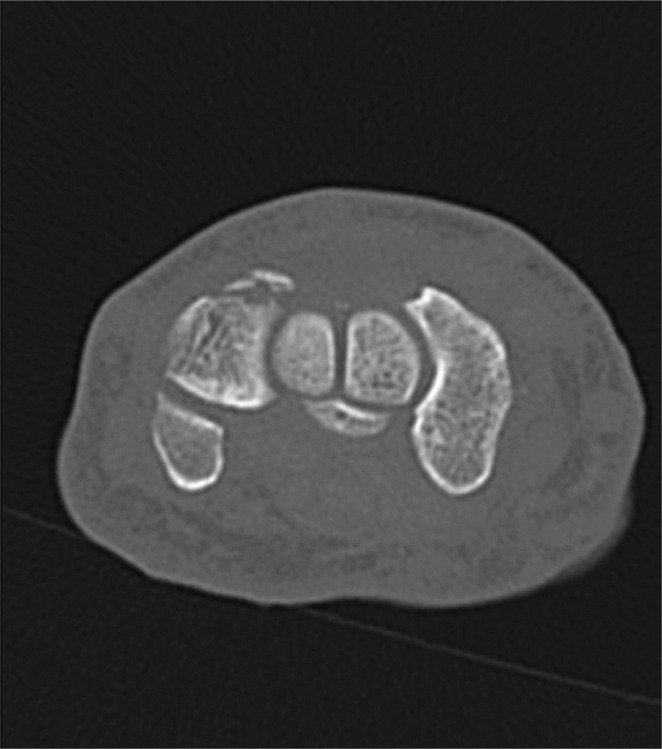
Typical CT scan finding in axial reconstruction

**Fig. 4:**
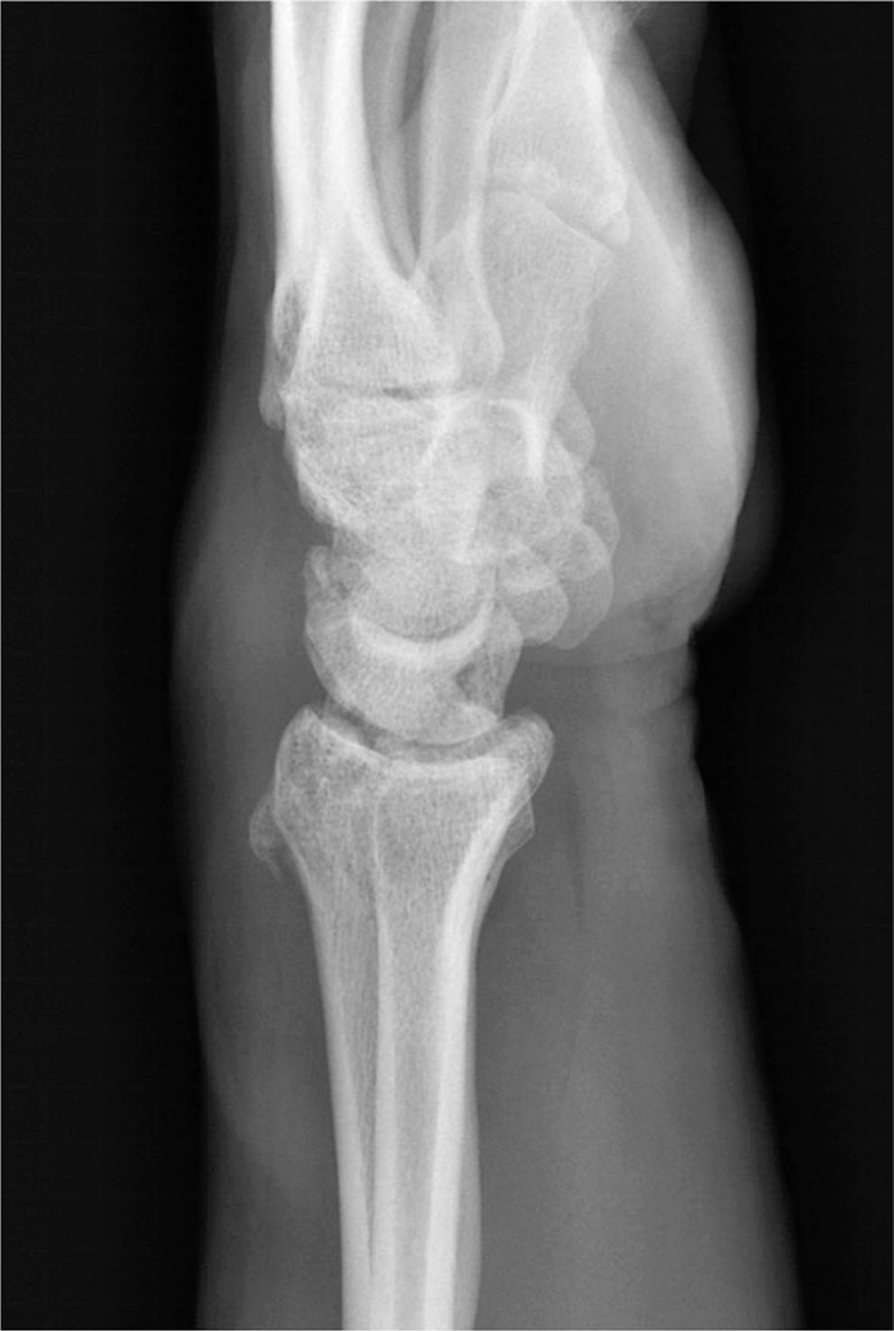
X- ray of dorsal triquetrum chip fractures combined with a distal radius fracture

The presented study focuses on dorsal triquetrum chip fractures since they are the most common triquetrum fracture type. Typical X-ray and CT-scan images are shown below (Figs. 1, 2 and 3). Figure 4 shows a dorsal triquetrum chip fracture combined with a distal radius fracture. The largest case series focusing exclusively on this fracture type is that of Höcker and Menschik from 1994 [3]. Most of the recent work- published by Vigler et al. [4], Suh et al. [6], Christie and Michelotti [8] and Guo et al. [2] each refers to this primary publication. Recommendations on conservative therapy varies and is not specified in detail. Suh and co-authors recommend casting between 3 and 4 weeks to allow optimal healing of the dorsal ligaments [6]. Similarly, Christie and Michelotti recommend cast therapy for 4–6 weeks [8]. Vigler et al. also recommend cast therapy or application of a splint for 4–6 weeks [4].

Overall, the data on dorsal cortical triquetral fractures is relatively sparse. To our knowledge, there is no recent literature focusing on the type of therapy and patient reported outcome.

The aim of this study is to comparatively analyse the outcomes of dorsal triquetrum chip fractures treated conservatively with a special focus on immobilisation modality (pre-fabricated wrist cuff or cast) and immobilisation duration. In addition, we compare isolated and combined injuries with regard to frequency and outcomes. Outcomes are evaluated by patient reported scores (PRWE and quick DASH), as well as, range of motion (ROM). The null hypothesis is that a cast immobilisation is superior to treatment with a prefabricated wrist splint with significant differences in clinical outcomes. Also, we hypothesize that conservative treatment results in good overall PROMs and range of motion. Furthermore, the duration of immobilisation (less than 6 weeks, 6 weeks or longer), the incidence and patient reported outcomes in isolated dorsal triquetrum chip fractures versus the presence of multiple lesions of ipsilateral hand, wrist and elbow were studied.

## Ethics

This retrospective cohort study was approved by the local ethics commission board. Recruited patients provided written informed consent.

## Materials and methods

### Study cohort and data collection

We identified all patients treated for dorsal triquetrum chip fractures between 2016 and 2020 at two public hospitals, by searching the electronic patient records.

Hospital charts of all identified patients were reviewed and data collected. In particular, we gathered type and duration of treatment, presence of a concomitant injury to the ipsilateral upper extremity (hand, wrist and elbow), active Range of motion at the end of treatment. The primary endpoint was defined as patient reported outcome (PROM) measured by PRWE (Patient Rated Wrist Evaluation) and Quick DASH (Disability of Arm, Shoulder and Hand). Both scores are well known and accepted to evaluate outcome after upper limb injuries. As secondary endpoint wrist range of motion at final clinical follow up was used.

### Treatment algorithm

Both study sites are mid-sized public hospitals located in the same state in two equally populated towns with a similar socio-economic profile. Patients were seen on the emergency ward or outpatient hand clinic. Different treatment options for dorsal cortical triquetrum chip fractures, using either a forearm cast or a prefabricated wrist cuff, were offered. The treatment decision was made by the on-call or advising hand surgeon based on the preference of the respective surgeon. This surgeon-specific approach resulted in two distinct treatment groups, creating a quasi-randomization of treatment modalities. This study leveraged this quasi-randomized setup to analyze the effect of immobilization type. Patients were then regularly followed-up in the hand surgery outpatient clinic. After a period of immobilisation, the wrist orthosis or cast was removed to begin free range of motion and weight bearing as tolerated. Patients were dismissed after obtaining normal wrist mobility, good function and absence of pain.

### Data analysis

Owing to the overall sample size, non-parametric tests were used. The significance level was set at 0.05 and the results are reported as medians and range if not stated otherwise. A paired t-test sample size estimation yielded a group size of 20 patients (alpha 0.05, power 0.8). Demographic variables were compared and outcomes were evaluated by PRWE, Quick DASH score and range of motion. A subgroup comparison was performed for the following:

1. Patients treated with a closed forearm cast vs. prefabricated wrist cuff.
2. Patients with isolated dorsal triquetrum chip fracture vs. with another concomitant ipsilateral injury of the hand, wrist and elbow.
3. Patients immobilised shorter than 6 weeks vs. immobilisation equal or longer to 6 weeks.

Numeric and categorical variables were analysed by Wilcoxon Rank Sum Test and Fisher’s exact test, respectively. Multiple comparison was performed by the Kruskal-Wallis test. Statistical analyses were computed using Stata/IC 15.1 software (StataCorp LP, College Station, TX, USA). This study adhered to the strengthening the reporting of observational studies in epidemiology reporting guidelines.

## Results

### Overall population and outcomes

Between 2016 and 2020 a total of 52 Patients were treated for dorsal triquetrum cortical chip fractures at the two sites. Three patients were underage and excluded. Furthermore, eight patients could not be contacted or were not willing to participate in questioning or were finally treated in another institution and are therefore lost to follow up. Another two patients were excluded from statistical analysis due to lack of wrist immobilisation (one patient had no cuff/cast and one was immobilised in a metacarpal brace). The final cohort consisted of 39 patients and wrists.

Of the 39 patients 11 patients were females and 28 males. Mean age was 48 years (median 52 years, range 22–76 years). 21 patients were treated with a prefabricated wrist cuff and 18 in a closed forearm cast. Median time of immobilisation was 6 weeks (range 3–9 weeks). 28 cases had an isolated dorsal cortical chip fracture, in 11 cases another ipsilateral hand, wrist and elbow lesion was found. Those include three intraarticular distal radius fractures, one extraarticular distal radius fracture, two pisiform fractures, one metacarpal fracture, one partial palmar plate rupture of a PIP finger joint, one radial head fracture, one peripheral TFCC lesion, one partial rupture of volar SL ligament. Median follow up in the outpatient clinic was 7 weeks (range 1–49 weeks, mean 9.38). Median follow from injury up to questioning for quick DASH and PRWE score was 38 months (range 3–64).

Overall, the mean PRWE score was 7.0 (median 0, range 0-48.5), mean quick DASH score was 5.8 (median 0, range 0–43). At final follow up, mean wrist flexion was 57° (median 60°, range 10–85°), mean extension 63° (median 65°, range 10–90°). Mean pronation was 83° (median 90, range 60–90°), mean supination 74° (median 80°, range 35–90°). Results are summarised in Table 1.

**Table 1:** Summary of demographics and overall outcome

	Median	Range	
		Min n	Max
Demographics			
Age (y)	52	22	76
Female	28%	11	
FUP clinical (weeks)	8	1	49
FUP score (weeks)	38	3	64
Immobilisation time (weeks)	6	3	9
Cast immobilisation	46%	18	
Concomitant injury	28%	11	
Outcomes			
PRWE	0	0	49
Quick Dash	0	0	43
Flexion Wrist (°)	60	10	85
Extension Wrist (°)	65	10	90
Arc Flexion-Extension (°)	130	40	170
Pronation Wrist (°)	90	60	90
Supination Wrist (°)	80	35	90
Arc Pro-Supination (°)	160	110	180

For variables female, immobilisation, concomitant injury percentages and count are reported. FUP denotes follow up. Only wrist range of motion is reported

### Treatment modality

To compare the treatment modality without the influence of concomitant injuries, only patients without further ipsilateral lesions of the upper limb were analysed. Hence, 28 patients remained to compare immobilisation in a prefabricated wrist splint and cast. Of those 28 patients 12 were treated with a prefabricated wrist cuff (group cuff) and 16 with a forearm cast (group cast). The gender distribution was similar: 13 males (81%) in the cuff group and 10 (83%) in the cast group. Median age in the cuff group was 54 years (28–76), median immobilisation time was 6 weeks (3–7). In the cast group, median age was 31 years (22–62), and median immobilisation time was 6 weeks (4–8). Median follow-up to the last visit in the outpatient clinic was 9 weeks (3–12) week in the cuff group, and 7 (1–49) weeks in the cast group. Median follow from accident to questioning for quick DASH and PRWE score was 26 months (3–64) and 38 months (23–58) for the cuff and cast group, respectively. Patients in the cast group were significantly younger compared to the cuff group (*p* = 0.0166). Otherwise, the demographic and other parameters did not show any significant differences. Results are summarised in Table 2.

**Table 2:** Summary of results according to immobilisation type (Cuff and Cast)

	Cuff			Cast				
	Median	Range		Median	Range		Diff.	P-val
		Min	Max		Min	Max		
Age (y)	54	28	76	31	22	62	23	0.0166
Female	19%	3		17%	2		2.1%	0.8888
FUP clinical (w)	9	3	12	7	1	49	2	0.2323
FUP score (w)	26	3	64	38	23	58	−11	0.0858
Immobilisation (w)	6	3	7	6	4	8	0	0.4004
PRWE	0	0	25	0	0	21	0	0.6994
Quick Dash	0	0	20	0	0	27	0	0.6995
Flexion Wrist (°)	60	40	80	62	30	85	−2	0.6149
Extension Wrist (°)	70	50	80	70	40	90	0	0.6501
Arc Flex-Extension (°)	130	90	160	130	70	170	0	0.6544
Pronation Wrist (°)	90	70	90	80	70	90	10	0.8276
Supination Wrist (°)	80	60	90	70	60	90	10	0.6763
Arc Pro-Supination (°)	165	130	180	150	130	180	15	0.7974

For variables female and immobilisation percentages are reported. Diff. denotes difference, diff = x(cuff) - x(cast). ROM denotes wrist range of motion. P-values are calculated by Wilcoxon-rank-sum test and Fishers exact test for continuous and dichotomous variables, respectively

The primary and secondary outcomes were similar and good in both groups. Mean PRWE was 6.7 and 4.8 in the cuff and cast group, respectively. Mean DASH score was 6.2 in the cuff group and 4.5 in the cast group. In the cuff group the mean flexion and extension was 60° and 67°, while the corresponding values in the cast group were 61° and 68°. Mean pro- and supination was 85-0-76° in the cuff group and 84-0-75° in the cast group. Further, wrist range of motion parameters were similar as well between groups. There was no significant difference in any outcome parameter between the cuff and cast group.

### Concomitant injuries

28 patients had isolated dorsal triquetrum chip fractures, while 11 patients had concomitant ipsilateral hand, wrist, or elbow injuries (Table 1). Table 3 shows type and quantity of concomitant injuries. Table 4 exhibits the comparison between dorsal triquetrum chip fractures which were isolated and such with concomitant hand and wrist injuries. Median age of patients with isolated injury was 53 (range 22–76) and 5 (18%) were female, compared to a median age of 50 years (range 28–65) and 6 (55%) females in the concomitant injury group. There is no significant age difference between groups, while the concomitant injury group had significantly more females (*p* = 0.0443). Patients with isolated injury were treated in 43% (*n* = 12) with a forearm cast with a median immobilisation of 6 weeks (range 3–8). Patients with concomitant injuries were treated in a forearm cast in 55% (*n* = 6) and median duration of immobilisation was 7 weeks (range 6–9). Immobilisation was longer in the concomitant injury group (*p* = 0.0011), but immobilisation modality did not differ statistically significant. Clinical follow up did not differ statistically and was 7.5 and 8.4 weeks for the isolated and concomitant injury group, respectively. However, follow up to final score was longer in the combined group (49 vs. 33 months, *p* = 0.0148).

**Table 3:** Type and quantity of concomitant ipsilateral elbow, wrist, hand injuries

Type	*n*
Intraarticular distal radius fracture	3
Pisiform fracture	2
Extraarticular distal radius fracture	1
Metacarpal fracture	1
Partial palmar plate rupture (finger PIP joint)	1
Radial head fracture	1
Peripheral TFCC lesion	1
Partial rupture of volar SL ligament	1

**Table 4:** Comparison between isolated dorsal triquetrum chip fractures and such with concomitant hand and wrist injuries

	Isolated injury			Combined Injury				
	Median	Range		Median	Range		Diff.	P-val
		Min n	Max		Min n	Max		
Age (y)	53	22	76	50	28	65	3	0.9253
Female	18%	5		55%	6		−36.7%	0.0443
FUP clinical (w)	8	1	49	8	3	37	−1	0.8503
FUP score (w)	33	3	64	49	24	60	−17	0.0184
Immobilisation (w)	6	3	8	7	6	9	−1	0.0011
Cast immobilisation	0			1			0	0.7226
PRWE	0	0	25	4	0	49	−4	0.4015
Quick Dash	0	0	27	0	0	43	0	0.9588
Flexion Wrist (°)	60	30	85	45	10	80	15	0.0823
Extension Wrist (°)	70	40	90	55	10	70	15	0.0061
Arc Flex-Extension (°)	130	70	170	100	40	145	30	0.0201
Pronation Wrist (°)	90	70	90	85	60	90	5	0.9699
Supination Wrist (°)	80	60	90	70	35	85	10	0.2300
Arc Pro-Supination (°)	160	130	180	150	110	175	10	0.2797

For variables female and immobilisation percentages are reported. Diff. denotes difference, diff = x(isolated) - x(combined). ROM denotes wrist range of motion. P-values are calculated by Wilcoxon-rank-sum test and Fishers exact test for continunous and dichotomous variables, respectively

The isolated and concomitant group showed no significant difference in the PROMs with a mean PRWE of 5.8 (range 0–25) for isolated injuries and a mean Quick DASH of 5.5 (range 0–27). Patients with a concomitant injury had a mean PRWE of 9.9 (range 0–49) and a mean quick DASH of 6.5 (range 0–43).

Considering secondary endpoints, wrist flexion and extension was better in the isolated injury group (flexion 60°, extension 70°) when compared to the concomitant injury group (flexion 45°, extension 55°). However, only extension differed significantly (*p* = 0.0061). Pro-/Supination was similar in both groups and did not statistically differ.

### Duration of immobilisation

To analyse the effect of immobilisation time, only patients without further ipsilateral hand and wrist lesions were analysed, yielding 28 patients which were divided in two groups: Patients immobilised for less than 6 weeks (short immobilisation group) and such with an immobilisation for 6 weeks or more (long immobilisation group). Only four patients where immobilised for less than 6 weeks, while 24 had a longer immobilisation. In the short immobilisation group, the duration of splinting/casting was 3 weeks for one patient, 4 weeks for two patients and 5 weeks for one patient. In the long immobilisation group, 22 patients were splinted for 6 weeks, while one was immobilised 7 weeks and one 8 weeks. The uneven group sizes limits the comparison. Results for this subgroup analysis are reported in Table 5.

**Table 5:** Subgroup analysis and results according to immobilisation time

	Immobilisation < 6weeks			Immobilisation > = 6 Weeks				
	Median	Range		Median	Range		Diff.	P-val
		Min	Max		Min	Max		
Age	53	32	65	53	22	76	0	0.7174
Female	0%	0		21%	5		−21%	0.3226
FUP clinical (w)	6	4	7	8	1	49	−3	0.0850
FUP score (w)	33	28	50	34	3	64	−2	0.7427
Cast immobilisation	75%	3		38%	9		38%	0.1683
PRWE	0	0	21	0	0	25	0	0.6361
Quick DASH	0	0	20	0	0	27	0	0.6889
Flexion Wrist (°)	61	60	70	60	30	85	1	0.7807
Extension Wrist (°)	65	56	80	70	40	90	−5	0.7014
Arc Flex-Extension (°)	125	118	150	130	70	170	−5	0.8365
Pronation Wrist (°)	90	80	90	80	70	90	10	0.2749
Supination Wrist (°)	75	70	80	80	60	90	−5	0.7727
Arc Pro-Supination (°)	165	150	170	160	130	180	5	0.8037

For variables female and immobilisation percentages are reported. Diff. denotes difference, diff = x(< 6w) - x(> = 6w). ROM denotes wrist range of motion. P-values are calculated by Wilcoxon-rank-sum test and Fishers exact test for continuous and dichotomous variables, respectively.

Age was the same with a mean 52.5 in both groups, while the short immobilisation group had no females and the long one had five female patients. Immobilisation modality, follow up clinical and score did not significantly differ between groups.

With regard to PROMs both groups were also similar and good. Mean PRWE/quick DASH was 5.1/5.0 and 6.0/5.5 for the short and long immobilisation groups, respectively. The difference was not statistically relevant. For the secondary outcome, a slight trend toward greater range of motion in the short immobilisation group was spotted. However, this difference was not significant, which probably is due to the sample size.

## Discussion

In this retrospective cohort study with quasi randomised immobilisation modality, it could be found, that dorsal triquetrum chip fractures can be treated non-operatively with excellent patient reported and clinical outcomes. There was no significant difference between type and time of immobilisation. Ipsilateral concomitant hand and wrist injuries yielded worse clinical outcomes with a trend to less favourable patient-reported outcomes, the latter without significant differences.

These findings are comparable to the series by Höcker and Menschik published in 1994 and is the only study to date investigating a patient cohort with dorsal triquetrum chip fractures. They included 231 triquetrum fractures of which 93% were dorsal triquetrum chip fractures, however, clinical and radiological follow-up was only available for 65 patients. An unstandardized cast immobilization was applied for a mean of 29.7 days. Höcker and Menschik found 15.6% concomitant injuries [3]. In our patient cohort concomitant ipsilateral injuries were more frequent with 27%. One explanation might be the more frequent use of CT-scans in modern medicine and, thus, more injuries might be detectable. Höcker and Menschik described the outcomes in 86% as very good, 12.5% as good and in 1.5% as poor (in one patient with a concomitant TFCC lesion), yet provides no definition for the outcome measure [3]. However, more detailed outcomes like pain, range of motion or patient reported outcomes are missing in their study. The presented study is to our knowledge the first so far to analyse patient reported outcome measures. A mean PRWE score of 7.1 and mean quick DASH score of 5.7 in our study cohort shows good results as well. In particular these scores compare favourably to the mean PRWE score of 7.7 and quick DASH score between 9 and 27 for healthy individuals in two large scale surveys [9, 10].

Vigler et al. [4], Suh et al. [6] and Christie and Michelotti [8] did not examine their own patients but instead referred to the patient cohort of Höcker and Menschik [3]. Suh et al. recommended 3 to 4 weeks for dorsal triquetrum chip fractures [6] whereas Christie and Michelotti advocate for 4 to 6 weeks of (not specified) immobilisation [8].

Guo et al. suggest 4 weeks of cast and 2 weeks of splint immobilisation [2] again with no patient cohort of their own, but referring indirectly to those of Höcker and Menschik. Mahmood and Lee believe that dorsal triquetrum chip fractures are comparable to soft tissue injuries and advises early return to sports with a splint [11]. They again did not research an own patient cohort but rely on those of Höcker and Menschik [3], Papp [1] and Geissler [12] – who both refers as well to Höcker and Menschik.

A study by Heo et al. examined the frequency of carpal fractures in combination with operatively treated radius fractures. In 223 distal radius fractures they found 46 (20.6%) concomitant carpus fractures, of which the most common were triquetrum fractures with 23 (10.3%) and of these 12 (5.4%) dorsal triquetrum chip fractures. All carpal fractures except one scaphoid fracture were treated non-operatively. All distal radius fractures were treated with open reduction, internal fixation and 4 weeks of splinting [13]. Hence, the non-operative treatment of dorsal triquetrum chip fractures in this patient cohort is comparable to our findings.

In our final cohort, in 11 out of 39 cases another ipsilateral hand, wrist and elbow lesion was found.

Those include three intraarticular – and one extraarticular distal radius fractures, two pisiform fractures, one metacarpal fracture, one partial palmar plate rupture of a PIP finger joint, one radial head fracture, one peripheral TFCC lesion, one partial rupture of volar SL ligament (the latter both diagnosed by MRI). Three out of those eleven patients showed ipsilateral carpal injuries (2 pisiform fractures, 1 partial rupture of volar SL ligament). Associated carpal injuries could significantly influence treatment especially in case of displaced carpal bone fractures or ligament injuries with carpal instability. Triquetral body fractures can be a feature of peri lunate fracture dislocation injuries or peri lunate injuries not dislocated (PLIND injuries). We therefore recommend low threshold to further imaging modalities such as CT scan in case of suspected additional carpal bone fracture and involvement of triquetral body and dynamic X-Ray (cinematography) or MRI in suspected ligamentous injury. We consider this mandatory in cases with atypical presentation and history such as high energy trauma mechanism, important swelling/severe pain, much more displaced dorsal or large chip fragment or radiological signs of carpal instability (scapho-lunate and luno-triquetral diastasis, malalignment with DISI/VISI position and interrupted carpal arcs (Gilula’s lines).

Our study is limited by its retrospective character. Furthermore, only four patients with an isolated dorsal triquetrum chip fracture were immobilised for 4 weeks or less, which makes it unfeasible to provide reliable recommendations for short-term immobilisation. Subgroups (immobilization time < 6 weeks and combined injury group) are underpowered in our study. To better compare treatment modalities and outcome (immobilisation time 6 weeks vs. immobilization < 6 weeks, patients with concomitant injuries) with small effect size (5–7 points difference in PRWE/QuickDASH score) and power 80% (α = 0.05) approximately 40–50 patients per group are necessary resulting in at least 120 total patients needed in three treatment arms. Future studies should meet at least these parameters to have enough power to distinguish between the subgroups.

Another limitation is that the patients treated with a cast were significantly younger than those treated with a splint. Otherwise, there is no difference in demographics. Since this study is retrospective, there was no way to influence the difference in age between the groups. The quasi-randomization of using either a cast or a splint—resulting from differing treatment regimens at the preference of the involved surgeon—was utilized to compare different treatment modalities. It shows the uncertainty of the correct treatment for this entity of dorsal chip fractures of the triquetrum. Surgeons from different institutions or clinical backgrounds tend to manage these fractures differently. Although our quasi-randomization does not match the rigor of a prospective randomized controlled trial, it nonetheless offers stronger protection against bias than treatment decisions based solely on individual patient specific circumstances, such as pain or swelling at presentation.

Our study does not have a control group with no immobilisation. This is due to the retrospective study design and potential difficulties to get ethics approval for the control group, since it differs from the general standard to treat fractures. Though, it would be very interesting to learn more about the outcomes in this control group without any or a very short time of immobilisation. We suggest a prospective multi-centre-controlled trial to further answer this question.

## Conclusion

Dorsal triquetrum chip fractures can be treated non-operatively with a simple prefabricated wrist splint for 4 to 6 weeks and yield excellent patient reported and clinical outcomes.

## Data Availability

No datasets were generated or analysed during the current study.

## References

[1] Papp S. Carpal bone fractures. Hand Clin. 2010;26:119–27. 20006250 10.1016/j.hcl.2009.08.014

[2] Guo RC, Cardenas JM, Wu CH. Triquetral fractures overview. Curr Rev Musculoskelet Med. 2021;14:101–6. 33483875 10.1007/s12178-021-09692-w PMC7991068

[3] Höcker K, Menschik A. Chip fractures of the triquetrum. Mechanism, classification and results. J Hand Surg Br. 1994;19:584–8. 7822914 10.1016/0266-7681(94)90120-1

[4] Vigler M, Aviles A, Lee SK. Carpal fractures excluding the scaphoid. Hand Clin. 2006;22:501–16. 17097470 10.1016/j.hcl.2006.07.007

[5] Garcia-Elias M. Dorsal fractures of the triquetrum-avulsion or compression fractures? J Hand Surg Am. 1987;12:266–8. 3559084 10.1016/s0363-5023(87)80285-x

[6] Suh N, Ek ET, Wolfe SW. Carpal fractures. J Hand Surg Am. 2014;39:785–91. 24679911 10.1016/j.jhsa.2013.10.030

[7] Gan LP, Satkunanantham M, Sreedharan S, et al. Triquetral fracture with associated pisiform subluxation. Singap Med J. 2015;56:39–41. 10.11622/smedj.2015049 PMC4371207 25820858

[8] Christie BM, Michelotti BF. Fractures of the carpal bones. Clin Plast Surg. 2019;46:469–77. 31103090 10.1016/j.cps.2019.03.007

[9] Aasheim T, Finsen V. The DASH and the quickdash instruments. Normative values in the general population in Norway. J Hand Surg Eur Vol. 2014;39:140–4. 23520389 10.1177/1753193413481302

[10] Mulders MAM, Kleipool SC, Dingemans SA, et al. Normative data for the patient-rated wrist evaluation questionnaire. J Hand Ther. 2018;31:287–94. 29132647 10.1016/j.jht.2017.10.007

[11] Mahmood B, Lee SK. Carpal fractures other than scaphoid in the athlete. Clin Sports Med. 2020;39:353–71. 32115089 10.1016/j.csm.2019.12.006

[12] Geissler WB. Carpal fractures in athletes. Clin Sports Med. 2001;20:167–88. 11227704 10.1016/s0278-5919(05)70254-4

[13] Heo YM, Kim SB, Yi JW, et al. Evaluation of associated carpal bone fractures in distal radial fractures. Clin Orthop Surg. 2013;5:98–104. 23730472 10.4055/cios.2013.5.2.98 PMC3664678

